# Case Commentary: When susceptibility is not enough in recurrent *Burkholderia cenocepacia* infection

**DOI:** 10.1128/aac.00467-26

**Published:** 2026-05-28

**Authors:** Simone Giuliano, Paolo Gaibani, Carlo Tascini

**Affiliations:** 1 Infectious Diseases Unit, Azienda Sanitaria Universitaria Friuli Centrale 18026 , Udine, Italy; 2 Department of Diagnostic and Public Health, Microbiology Section, Verona University 19051, Verona, Italy; 3 Microbiology and Virology Unit, Department of Pathology, Azienda Ospedaliera Universitaria Integrata Di Verona 9286, Verona, Italy; 4 Department of Medicine, DMED University of Udine 196018, Udine, Italy; Houston Methodist Hospital and Weill Cornell Medical College, Houston, Texas, USA

**Keywords:** *Burkholderia cenocepacia*, cefiderocol, sulbactam-durlobactam, β-lactam combination therapy, penicillin-binding proteins, cystic fibrosis, antimicrobial susceptibility testing

## Abstract

In this issue, B. Gaglani, T. M. Andermann, C. Wate, J. Oldan, et al. (Antimicrob Agents Chemother 70:e01542-25, 2026, https://doi.org/10.1128/aac.01542-25) describe a recurrent *Burkholderia cenocepacia* infection successfully treated with cefiderocol plus sulbactam-durlobactam. Treatment decisions are often made with incomplete microbiological and pharmacological data, even as interest in mechanism-based β-lactam combination strategies continues to grow. This case supports the utility of cefiderocol plus sulbactam-durlobactam in *B. cenocepacia* infection and reinforces the importance of multidisciplinary approaches to better estimate the risk of treatment failure and optimize antimicrobial therapy.

## COMMENTARY

In this issue, Gaglani and colleagues (1) describe a clinically complex case of recurrent intra-abdominal infection due to *Burkholderia cenocepacia* in a lung transplant recipient with cystic fibrosis, presenting as perihepatitis consistent with Fitz-Hugh-Curtis syndrome. Although this report raises important clinical considerations regarding the potential role of the sulbactam-durlobactam (SUL-DUR) and cefiderocol (FDC) combination in the treatment of *B. cenocepacia* infections, its broader value lies in illustrating a recurring dilemma in contemporary antimicrobial decision-making.

First, treatment decisions in difficult-to-treat (DTR) infections caused by multidrug-resistant (MDR) pathogens with limited therapeutic options are frequently made in the setting of incomplete microbiological information, major host-related constraints, and a narrow therapeutic margin. Although clinical improvement in the reported case followed source control, adjustment of immunosuppressive therapy, and treatment with FDC plus SUL-DUR, the absence of pharmacological and microbiological data raises important concerns about the optimal framework for guiding antibiotic therapy in severe DTR infections. Indeed, factors such as renal dysfunction, altered pharmacokinetics, and the presence of a deep-seated infection may substantially influence outcomes, highlighting the need to interpret antimicrobial efficacy within its broader therapeutic, microbiological, and pharmacological context.

Second, the report also highlights a key limitation of selecting β-lactam combinations without synergy testing data. This issue has become increasingly relevant as the number of available β-lactam and β-lactamase inhibitor combinations has expanded, while clinically validated tools to guide combination selection remain limited (2–4). Indeed, although several clinical studies suggest that combination therapy may improve survival in patients with DTR infections caused by MDR pathogens, major uncertainties remain regarding the optimal choice of agents and the appropriate modification of treatment in cases of non-response or relapse. In the ATTACK trial, SUL-DUR was administered together with imipenem-cilastatin for the treatment of severe *Acinetobacter baumannii* complex infections, and subsequent microbiological and structural analyses helped clarify the rationale for that backbone (5, 6). Specifically, these data supported complementary engagement of different penicillin-binding proteins (PBPs), with sulbactam contributing activity against PBP1a, PBP1b, and PBP3, and imipenem providing strong activity against PBP1a and PBP2 (6). This framework is consistent with more recent observations in carbapenem-resistant *A. baumannii*, in which FDC plus ampicillin-sulbactam retained activity despite PBP alterations (7). A similar target-based interpretation was also proposed in a recent case report describing successful treatment of NDM-producing *Escherichia coli* carrying a PBP3 alteration with imipenem-relebactam plus aztreonam (8). Taken together, these observations support the hypothesis that β-lactam combinations may act synergistically at multiple levels, including β-lactamase inhibition and complementary engagement of PBPs. In the present case, the *B. cenocepacia* isolate showed a progressive increase in the ceftazidime-avibactam MIC. It is conceivable that this reflected, at least in part, alterations involving PBP3, which may also have influenced FDC activity. Indeed, PBP3 alterations have been associated—together with other mechanisms, including efflux pump changes and β-lactamase production—with reduced FDC susceptibility in *Pseudomonas aeruginosa*, *E. coli*, and *A. baumannii* (9–12). In this context, target redundancy and complementary PBP occupancy may have contributed to the clinical benefit observed with FDC plus SUL-DUR in *B. cenocepacia*. FDC primarily targets PBP3 in gram-negative bacteria, including *E. coli* (13); SUL has intrinsic binding activity against PBP1a and PBP3 in *E. coli* (14); and DUR, in addition to inhibiting serine β-lactamases of Ambler classes A, C, and D (15), has shown high affinity for PBP2 in *E. coli* (16). Given that *B. cenocepacia* encodes homologs of the *E. coli* PBP1a, PBP2, and PBP3 systems (17–19), it is plausible that this combination achieved complementary PBP engagement, thereby enhancing bactericidal activity.

Third, it is also reasonable to ask whether imipenem-relebactam might also have represented a plausible combination partner, given its mechanistic appeal in the *B. cepacia* complex, where class A and class C β-lactamases coexist, and its retained *in vitro* activity against a substantial proportion of ceftazidime-avibactam-resistant isolates (8, 20, 21). The possible contribution of imipenem should also be considered carefully. In the SUL-DUR development program, imipenem was the companion drug used in the pivotal trial, a design that may have offered advantages beyond copathogen coverage by increasing PBP target redundancy and broadening inhibition across complementary cell-wall targets (5, 22). Ferretti and colleagues showed that cefiderocol has antibiofilm activity against *P. aeruginosa* and that its combination with imipenem increased bactericidal activity against biofilm-grown cells compared with FDC alone (23). These findings cannot be directly extrapolated to *B. cenocepacia*, but they support the possibility that complementary target inhibition may matter in biofilm-associated infection as much as in planktonic growth.

Fourth, additional laboratory points deserve attention. The absence of therapeutic drug monitoring for ceftazidime-avibactam is clinically relevant, especially in patients with renal dysfunction, where insufficient monitoring may contribute to both target non-attainment and neurotoxicity related to cephalosporin overexposure (24, 25). At the same time, genomic characterization should be considered an integral component of antimicrobial stewardship, as it may help estimate the risk of treatment failure based on the resistome profile and provide prognostic information for the management of infections caused by pathogens with similar resistance patterns (26, 27).

In conclusion, this case supports the potential therapeutic utility of the CFD–SUL-DUR combination in a *B. cenocepacia* infection and underscores the need for multidisciplinary decision-making integrating pharmacological, microbiological, and clinical factors to optimize antimicrobial therapy for DTR infections caused by MDR pathogens.

## References

[1] Gaglani B, Andermann TM, Wate C, Oldan J, Desai CS, Marshall SH, Bonomo RA, Coakley R, van Duin D, Lachiewicz A. 2026. Sulbactam-durlobactam and cefiderocol combination treatment of Burkholderia cenocepacia-associated Fitz-Hugh-Curtis syndrome. Antimicrob Agents Chemother:e0154225. doi:10.1128/aac.01542-25 42059812 PMC13321792

[2] Doern CD. 2014. When does 2 plus 2 equal 5? A review of antimicrobial synergy testing. J Clin Microbiol 52:4124–4128. doi:10.1128/JCM.01121-14 24920779 PMC4313275

[3] Brennan-Krohn T, Kirby JE. 2019. When one drug is not enough. Clin Lab Med 39:345–358. doi:10.1016/j.cll.2019.04.002 31383261 PMC6686866

[4] Tang P-C, Sánchez-Hevia DL, Westhoff S, Fatsis-Kavalopoulos N, Andersson DI. 2024. Within-species variability of antibiotic interactions in gram-negative bacteria. mBio 15:e0019624. doi:10.1128/mbio.00196-24 38391196 PMC10936430

[5] Kaye KS, Shorr AF, Wunderink RG, Du B, Poirier GE, Rana K, Miller A, Lewis D, O’Donnell J, Chen L, Reinhart H, Srinivasan S, Isaacs R, Altarac D. 2023. Efficacy and safety of sulbactam-durlobactam versus colistin for the treatment of patients with serious infections caused by Acinetobacter baumannii-calcoaceticus complex: a multicentre, randomised, active-controlled, phase 3, non-inferiority clinical trial (ATTACK). Lancet Infect Dis 23:1072–1084. doi:10.1016/S1473-3099(23)00184-6 37182534

[6] Veeraraghavan B, Shin E, Bakthavatchalam YD, Manesh A, Dubey D, Tascini C, Taracila MA, Hujer AM, Jacobs MR, Bonomo RA. 2025. A microbiological and structural analysis of the interplay between sulbactam/durlobactam and imipenem against penicillin-binding proteins (PBPs) of Acinetobacter spp. Antimicrob Agents Chemother 69:e0162724. doi:10.1128/aac.01627-24 40035550 PMC11963609

[7] Tascini C, Giuliano S, Martini L, Carannante N, Mariano B, Perilli M, Piccirilli A. 2025. OXA-carbapenemases and mutations within PBPs in ST2 carbapenem-resistant A. baumannii: evaluating the efficacy of cefiderocol and ampicillin-sulbactam combination therapy. J Glob Antimicrob Resist 44:189–196. doi:10.1016/j.jgar.2025.06.014 40582404

[8] Fabrizio C, Valzano F, Giuliano S, Morelli E, Serio D, Buccoliero GB, Cervellera M, La Bella G, Arena F, Hujer AM, Taracila MA, Marshall SH, Bonomo RA, Tascini C. 2026. Optimizing target inactivation to treat multidrug-resistant Escherichia coli with NDM and PBP3 mutations: “going the extra mile”. Antimicrob Agents Chemother 70:e0088725. doi:10.1128/aac.00887-25 41718487 PMC13148025

[9] Oliver A, Arca-Suárez J, Gomis-Font MA, González-Pinto L, López-Causapé C. 2025. Emerging resistance mechanisms to newer β-lactams in Pseudomonas aeruginosa. Clin Microbiol Infect 31:1790–1796. doi:10.1016/j.cmi.2025.03.013 40120758

[10] Gomis-Font MA, Sastre-Femenia MÀ, Taltavull B, Cabot G, Oliver A. 2023. In vitro dynamics and mechanisms of cefiderocol resistance development in wild-type, mutator and XDR Pseudomonas aeruginosa. J Antimicrob Chemother 78:1785–1794. doi:10.1093/jac/dkad172 37253034

[11] Livermore DM, Mushtaq S, Vickers A, Woodford N. 2023. Activity of aztreonam/avibactam against metallo-β-lactamase-producing Enterobacterales from the UK: impact of penicillin-binding protein-3 inserts and CMY-42 β-lactamase in Escherichia coli. Int J Antimicrob Agents 61:106776. doi:10.1016/j.ijantimicag.2023.106776 36893810

[12] Malik S, Kaminski M, Landman D, Quale J. 2020. Cefiderocol resistance in Acinetobacter baumannii: roles of β-lactamases, siderophore receptors, and penicillin binding protein 3. Antimicrob Agents Chemother 64:e01221-20. doi:10.1128/AAC.01221-20 32868330 PMC7577126

[13] Ito A, Sato T, Ota M, Takemura M, Nishikawa T, Toba S, Kohira N, Miyagawa S, Ishibashi N, Matsumoto S, Nakamura R, Tsuji M, Yamano Y. 2018. In vitro antibacterial properties of cefiderocol, a novel siderophore cephalosporin, against gram-negative bacteria. Antimicrob Agents Chemother 62:e01454-17. doi:10.1128/AAC.01454-17 PMC5740388 29061741

[14] Kazmierczak A, Pechinot A, Siebor E, Cordin X, Labia R. 1989. Sulbactam: secondary mechanisms of action. Diagn Microbiol Infect Dis 12:139S–146S. doi:10.1016/0732-8893(89)90126-0 2591173

[15] Papp-Wallace KM, McLeod SM, Miller AA. 2023. Durlobactam, a broad-spectrum serine β-lactamase inhibitor, restores sulbactam activity against Acinetobacter species. Clin Infect Dis 76:S194–S201. doi:10.1093/cid/ciad095 37125470 PMC10150275

[16] Durand-Réville TF, Guler S, Comita-Prevoir J, Chen B, Bifulco N, Huynh H, Lahiri S, Shapiro AB, McLeod SM, Carter NM, et al.. 2017. ETX2514 is a broad-spectrum β-lactamase inhibitor for the treatment of drug-resistant gram-negative bacteria including Acinetobacter baumannii. Nat Microbiol 2:17104. doi:10.1038/nmicrobiol.2017.104 28665414

[17] Hogan AM, Rahman ASMZ, Motnenko A, Natarajan A, Maydaniuk DT, León B, Batun Z, Palacios A, Bosch A, Cardona ST. 2023. Profiling cell envelope-antibiotic interactions reveals vulnerabilities to β-lactams in a multidrug-resistant bacterium. Nat Commun 14:4815. doi:10.1038/s41467-023-40494-5 37558695 PMC10412643

[18] Chantratita N, Rholl DA, Sim B, Wuthiekanun V, Limmathurotsakul D, Amornchai P, Thanwisai A, Chua HH, Ooi WF, Holden MTG, Day NP, Tan P, Schweizer HP, Peacock SJ. 2011. Antimicrobial resistance to ceftazidime involving loss of penicillin-binding protein 3 in Burkholderia pseudomallei. Proc Natl Acad Sci USA 108:17165–17170. doi:10.1073/pnas.1111020108 21969582 PMC3193241

[19] Specht KM, Sheetz KH, Alexander CM, Lamech LT, O’Connor LH, Walker DM, Stevenson HP. 2010. Expression and characterization of penicillin-binding proteins in Burkholderia cenocepacia. Curr Microbiol 60:274–279. doi:10.1007/s00284-009-9537-1 19924480

[20] Becka SA, Zeiser ET, LiPuma JJ, Papp-Wallace KM. 2021. Activity of imipenem-relebactam against multidrug- and extensively drug-resistant burkholderia cepacia complex and Burkholderia gladioli. Antimicrob Agents Chemother 65:e0133221. doi:10.1128/AAC.01332-21 34370574 PMC8522746

[21] Campanella TA, Gallagher JC. 2020. A clinical review and critical evaluation of imipenem-relebactam: evidence to date. Infect Drug Resist 13:4297–4308. doi:10.2147/IDR.S224228 33268997 PMC7701153

[22] Giuliano S, Sbrana F, Tascini C. 2023. Sulbactam-durlobactam for infections caused by Acinetobacter baumannii-calcoaceticus complex. Lancet Infect Dis 23:e274. doi:10.1016/S1473-3099(23)00422-X 37442147

[23] Ferretti C, Poma NV, Bernardo M, Rindi L, Cesta N, Tavanti A, Tascini C, Di Luca M. 2024. Evaluation of antibiofilm activity of cefiderocol alone and in combination with imipenem against Pseudomonas aeruginosa. J Glob Antimicrob Resist 37:53–61. doi:10.1016/j.jgar.2024.01.021 38331031

[24] Gatti M, Viale P, Pea F. 2024. Therapeutic drug monitoring of ceftazidime/avibactam: why one leg is not enough to run. J Antimicrob Chemother 79:195–199. doi:10.1093/jac/dkad367 38019676

[25] Vanneste D, Gijsen M, Maertens J, Van Paesschen W, Debaveye Y, Wauters J, Spriet I. 2024. Ceftazidime-related neurotoxicity in a patient with renal impairment: a case report and literature review. Infection 52:1113–1123. doi:10.1007/s15010-023-02167-9 38305827

[26] Stracquadanio S, Nicolosi A, Privitera GF, Massimino M, Marino A, Bongiorno D, Stefani S. 2024. Role of transcriptomic and genomic analyses in improving the comprehension of cefiderocol activity in Acinetobacter baumannii. mSphere 9:e0061723. doi:10.1128/msphere.00617-23 38078714 PMC10826366

[27] Shelburne SA, Kim J, Munita JM, Sahasrabhojane P, Shields RK, Press EG, Li X, Arias CA, Cantarel B, Jiang Y, Kim MS, Aitken SL, Greenberg DE. 2017. Whole-genome sequencing accurately identifies resistance to extended-spectrum β-lactams for major gram-negative bacterial pathogens. Clin Infect Dis 65:738–745. doi:10.1093/cid/cix417 28472260 PMC5850535

